# Assessing the effectiveness of a digital, case-based learning platform for cancer pain management in residency training

**DOI:** 10.5116/ijme.6563.251d

**Published:** 2023-12-15

**Authors:** Saowanee Sawang, Rattaphol Seangrung, Nuj Tontisirin, Panita Wanpiroon

**Affiliations:** 1 Department of Anesthesiology, Faculty of Medicine Ramathibodi Hospital, Bangkok, Thailand; 2 Department of Education Technology and Information Science, Faculty of Technical Education, King Mongkut's Universi-ty of Technology North Bangkok, Bangkok, Thailand

**Keywords:** Cancer pain education, case-based learning, digital learning

## Abstract

**Objectives:**

To determine the effectiveness of self-directed, case-based learning in cancer pain management via a digital learning platform (e-CBL) in interdisciplinary residents' knowledge and critical thinking skill level.

**Methods:**

The prospective observational study was conducted on 51 first- and second-year residents from anesthesiology, surgery, and family medicine who had not received training in the management of cancer pain and were invited to participate by their Program Director. Participants voluntarily underwent e-CBL in cancer pain management using four modules (pain assessment, principles of pain management, pharmacological techniques, and non-pharmacological techniques) at their convenience within seven days via the Moodle platform. All participants underwent pre-and post-test assessments of knowledge and rated their satisfaction with the training on a 0-10 scale. Thirty-two residents completed Cornell Critical Thinking Test Level Z. Paired t-tests assessed changes, and the effect size was estimated by Cohen's d. A p-value < .05 was considered statistically significant.

**Results:**

Knowledge and critical thinking test results significantly improved after the training (M=68, SD=16.99 to M=86, SD=13.96 correct responses; t_(50)_=11.24, p<.001, Cohen's d=1.56 for knowledge) and (M=39.8, SD=13.7 vs. M=46.1, SD=10.2 correct responses; t_(31)_=-3.67, p=.001, Cohen's d=0.65 for critical thinking test). Satisfaction of learning experiences for convenience and understandability was high (M=9.4, SD=0.8).

**Conclusions:**

Use of the e-CBL improved knowledge in cancer pain management and critical thinking skills. This digital platform could play an important role in the future of pain education. Further investigation, including a control group, is warranted.

## Introduction

During the COVID-19 pandemic, many medical school classes were changed to case-based online learning because a limited number of patients were available and because of concerns regarding the students' safety.^1^ Additionally, many providers who would normally teach trainees were diverted to the provision of health care during the outbreak. A self-directed e-learning (SDL) platform allows students to learn at their own pace and convenience. Case-based learning (CBL) is a problem-based method that can help students connect knowledge and medical practice, develop professional skills, and enhance higher-order thinking.^2^  Compared to traditional teaching methods, CBL improves knowledge acquisition, problem-solving skills, and learner satisfaction.^3^ Moreover, SDL using CBL in graduate medical education improves knowledge, clinical skills and critical thinking skills to a similar degree as didactic teaching.^4^^-^^8^ A systematic review that included 51 studies of 6,750 students in medicine, nursing, dentistry, physical therapy and pharmacy showed that e-learning provides similar learning outcomes and satisfaction compared to traditional methods but is more flexible and cost-effective.^9^

Inadequate cancer pain management is a global problem despite increased awareness and initiatives, updated guidelines, and an increasing number of studies investigating its barriers. The rate of undertreatment of pain in cancer patients has not decreased substantially from 43.4% in 2016 to 40.0% in 2022.^10^ Moreover, a systematic review in 2016 that included 117 studies of 65,533 cancer patients demonstrated no significant change in the prevalence of cancer pain compared in the previous decade, with up to 38% of cancer patients reporting moderate-severe pain.^11^

In Asia, cancer pain is generally undermanaged. Factors that might explain this phenomenon are shortage of trained healthcare workers, and inadequate knowledge regarding assessment and management of cancer pain.^12^ A study of pain management in 10 countries in Asia demonstrated that more than half of physicians did not know how to prescribe pain medications properly, and they wanted more training to improve their knowledge and skills.^13^ Undertreatment of cancer pain in Thailand is well documented; 40% of hospitalized cancer patients experienced pain, and half of those who received pain medication had to request it.^14^ Chinda M and colleagues showed that a lack of knowledge of pain medications and inappropriate attitudes about pain were associated with poor pain management skills in general practitioners and residents in Thailand.^15^

Pain education for health professionals is repeatedly identified as a key to improving pain management. A comprehensive review of twenty-five years of research on pain education suggested moving forward to "advanced pain education", which must shift from a theoretical basis to a clinical environment where contextual decision-making is practised.^16^ A study of electronically delivered undergraduate pain education that included expert-facilitated collaboration online or face-to-face significantly improved pain knowledge.^17^ However, the effectiveness of pure e-learning, which is a more sustainable system of pain education for health professionals, has not been studied. The goal of this study was to assess the effectiveness of an SDL electronic platform on the management of cancer pain using a case-based approach in improving knowledge and critical thinking skills of first- and second-year trainees in anesthesiology, surgery and family medicine. The secondary outcome was to examine the residents' experiences and satisfaction.

## Methods

### Study design

The study was approved by the Ethics Committee of the Faculty of Medicine Ramathibodi Hospital, Mahidol University. The prospective observational study was conducted from February 2022 to June 2022 at the Faculty of Medicine Ramathibodi Hospital, Bangkok, Thailand.

### Participants

First and second-year residents from anesthesiology, surgery, and family medicine who had not received training in the management of cancer pain at the pain clinic or who had not undergone a pain rotation were eligible to enroll in the study, and they were invited to participate by their Program Director. Participation was voluntary, and all participants provided written informed consent. We aimed to enroll at least forty participants based on the suggestion of an expert in digital education (PW). A total of 51 residents (F: M; 30:21), aged M=28.11, SD=0.6 years, completed four modules of e-CBL and pre and post-test of knowledge including residents from the Department of Anesthesiology 28 (55%), Family Medicine 14 (25%) and Surgery 11 (20%). However, only 32 participants completed the knowledge pre-and post-tests and the Critical thinking tests (32/51; 63%).

#### E-learning platform design and development

The e-learning platform was designed by three pain specialists (RS, SS, NT) and one specialist in digital education (PW). It contained four learning modules. The contents of each module were assessed and refined by four senior pain specialists who worked at Ramathibodi Hospital. The Modules included: 1) Pain assessment in cancer patients (12.51 minutes); 2) Principles of pain management (1.59 minutes); 3) Pharmacological treatment (22.43 minutes); and 4) Non-pharmacological management (2.26 minutes). Each module had five sequential steps:1:) case presentation, 2) creating a problem list, 3) summary of recent knowledge or guideline or recommendation, 4) making a relevant plan and 5) summary and application.

To enhance attention during each module, participants underwent quizzes that consisted of 8 multiple-choice questions either in or between the learning module. Eight quizzes contained one question about 1) pain assessment, 2) the type of pain, 3) the principle of acute pain management, 4) the principle of chronic pain management, 5) the type of analgesics, 6) the conversion ratio of morphine to fentanyl, 7) opioid-induced constipation, and 8) non-pharmacological pain management technique.

#### The e-learning process

Each module was posted in the Moodle learning platform and was available for seven days after students started the first module. The residents could complete the learning module at their own pace on any web-compatible device, including a smartphone, tablet, or PC. If the students had a question, they could send a message to the web administrator directly via Line-application.

#### Learning outcome and user experience - Pain knowledge

Pre and post-tests consisting of 30 MCQs were used to evaluate knowledge. Then, the score was converted to percentage. All MCQs were evaluated by six pain specialists from different University hospitals and each had an index of consistency (IOC) of more than 0.6. The MCQs assessed pain assessment (3 questions), pain classification (2 questions), principles of pain management (2 questions), techniques of pain management (1 question), choices of analgesics (10 questions), opioid conversion (5 questions), opioid side effects (5 questions) and non-pharmacological techniques (2 questions).

#### Critical thinking skills

The Cornel Critical Thinking Test Level Z measures cognitive abilities in students above grade 11. The Thai version is validated (criterion-related validity - concurrent validity; 0.70 and reliability; 0.75),^18^ and is widely used to investigate critical thinking ability.^19^ The test consists of 52 MCQs, which assess six domains based on the Theory of Ennis and Millman: 1) Deductive reasoning (10 questions); 2) Semantic (11 questions); 3) Credibility (4 questions); 4) Inductive reasoning (13 questions); 5) Prediction in planning experiment (4 questions); and 6) Definition and identification of assumptions (10 questions). The student is given 50 minutes to complete the test. One point was given for each correct response. The residents took the Critical Thinking Test before beginning the modules, and within 14 days after completing them.

For the pre and post-tests and the critical thinking test. The Moodle program randomly changed the order of choices in MCQs each time a test was given.

#### Learning experiences

At the end of study participation, participants filled out a satisfaction survey via the Moodle platform (0-10; 0 = unlikely and 10 = extremely likely) including 1) accessibility and convenience, 2) appropriateness and understandability and 3) satisfaction with the digital learning platform. Respondents were also invited to comments or suggestions.

#### Statistical Analysis 

The SPSS version 18.0 was used for statistical analysis The descriptive data was presented as percentages, and continuous data as mean and standard deviation. Normality of the continuous data was assessed with the Kolmogorov-Smirnov test. A paired t-test was used to compare pre vs. post-test of both knowledge and critical thinking skills if the data had a normal distribution. The effect size was calculated to demonstrate the strength of differences between pair measures and estimated by Cohen's d. A p-value < .05 was considered statistically significant.

## Results

There was a normal distribution of scores for both the knowledge and critical thinking tests. The results of the pre and post-tests of knowledge and critical thinking were shown in Table 1. Overall, knowledge and critical thinking significantly improved after completing the electronically delivered instructional modules, with an extremely large effect size for knowledge and a medium effect size for critical thinking level. Table 2 showed changes in performance on the individual subscales of the critical thinking test, with significant improvement in deductive reasoning, semantics, inductive reasoning and definition and identification of assumptions, again with medium effect sizes.

Overall, the learning experience was rated at a high level, including accessibility and convenience (M=9.4, SD=1.0), appropriateness and understandability (M=9.5, SD= 0.8) and satisfaction with the digital learning platform (M=9.4, SD=0.9). There was a wide range of learning time (Mdn =3.9 hours, IQR=0.8, 165 hours). There was no significant correlation between learning time and change in knowledge score, r_(__52)_= -.08, p = .56 and Cornell critical thinking score, r_(52)_ = -.04, p = .78.

## Discussion

Our study demonstrated that use of the newly developed self-directed case-based e-learning platform in cancer pain management improved both knowledge and critical thinking skill levels in multidisciplinary residents with high satisfaction scores. Our findings are similar to other studies that demonstrate that online courses in medical education can increase knowledge and learning satisfaction and provide a low cost-effectiveness ratio.^20^ Compared with traditional lectures, Koth and colleagues showed that an e-learning module provided similar knowledge outcomes without compromising critical thinking skills in dental education.^21^ For cancer pain education, Leung YW and colleagues showed that a facilitator-led online educational intervention can significantly enhance not only knowledge and confidence but also clinical skills in nurses.^22^

Three components of our e-learning modules could explain our successful outcomes, including 1) an appropriate learning technique (e-CBL) and material that included well-selected cases and teaching materials, 2) appropriate active tools to increase student engagement such as quizzes in each module, and 3) the flexibility of e-learning that allowed students to learn at their own pace and at convenient times.

**Table 1:** Comparative analysis of pre-test and post-test scores in knowledge and critical thinking tests

Variable	n	Pre-test		Post-test		t	P	Cohen's d.
		Mean	SD	Mean	SD			
Definition, identification of assumption (10)	7.59	3.07	8.78	2.34	-2.75	.01	.48
Knowledge test (total score of 100)	51	68.43	16.99	86.77	13.96	11.24	<.001	1.58
Critical Thinking test (total score of 52)	32	39.75	13.75	46.12	10.17	-3.67	.001	.65

Knowledge test =30 MCQ (presented as percentage), Cornell Critical Thinking test-level Z = 52 MCQ (total score of 52), Effect size was estimated by Cohen's d.

**Table 2:** Subscales of the Critical Thinking Test (n = 32)

Subscale of critical thinking test (Total score)	Pre-test		Post-test		t(31)	P	Cohen's d.
	Mean	SD	Mean	SD			
Deductive reasoning (10)	8.16	2.36	9.13	1.64	-3.35	.02	.59
Semantics (11)	7.91	3.38	9.56	2.59	-3.90	<.001	.69
Credibility (4)	3.03	1.26	3.53	0.92	-2.98	.06	.53
Inductive reasoning (13)	10.10	3.71	11.72	2.41	-3.21	.03	.57
Prediction in planning (4)	2.97	1.33	3.41	1.13	-1.99	.06	.35

Cornell Critical Thinking test-level Z = 52 MCQ (total score of 52), Effect size was estimated by Cohen's d.

First, the five steps in each case-based module allowed the students to consider the patient's current and past medical history, generate a problem list, take into account existing knowledge, and form and apply a plan. We carefully developed the material in each module and verified it with four senior pain specialists. The unified theory of acceptance and use of technology (UTAUT) model specifies four aspects that enhance e-learning, including performance expectancy (learning material or education is applicable), effort expectancy (ease of use), social influence (other influence on individuals' engagement) and facilitating condition (availability of assisting resources).^23^ Well-prepared material that is relevant to daily practice could improve in performance expectancy.^24^ Moreover,  students enjoy CBL and think that it enhances their learning.^3^ Similarly, teachers prefer CBL because it engages and motivates students.^3^

Second, our learning platform included active learning tools in the form of quizzes in the modules and between the modules to increase students' engagement. Lau and colleagues demonstrated that a dynamic e-learning module (e.g. interactive learner-centric dynamic scenario-based education) was more enjoyable than a static module (e.g. video record of linear static education), which impacts engagement and satisfaction.^25^ Rossi and colleagues showed that an online course with active learning methods can improve critical thinking skills, motivation and altitudes in sciences, especially the activities that require the interaction of information, prediction and reasoning.^26^ Additionally, the case that we included who had severe cancer pain and suffering may increase the emotional engagement of learners.

Third, the flexibility of e-learning is suitable for students with variable learning styles. Bahrambeygi and colleagues showed that self-pacing improved nurses' knowledge regarding venous thromboembolism.^27^ Shikino and colleagues demonstrated that using e-learning videos provided higher diagnostic accuracy scores for funduscopic examinations compared to didactic lectures.^28^ Moreover, we used the Moodle e-learning platform, which is familiar to many residents, thus enhancing effort expectancy according to the UTAUT model.

Critical thinking is essential for students to evaluate the accuracy of information that they receive and to develop new ideas. Additionally, the ability to think critically is crucial in diagnosis and practice, for example, in the intensive care unit.^29^ There is a significant relationship between critical thinking and self-directed learning in higher education^30^^-^^32^ which is an essential prerequisite for lifelong education, continuing professional development or public health leadership.^33^ Importantly, our case-based e-learning tools improved the critical thinking skills of learners.

Self-directed case-based e-learning can be an effective tool during challenging times such as pandemics or when there is limited time or access to educational resources, for example, a limited number of patients. Residents can access e-learning easily via their tablet, smartphone, or computer and learn at their own pace and time. Participants in our study rated accessibility and convenience, appropriateness and understandability, and satisfaction with the digital learning platform as highly satisfactory, enhancing the effort and performance expectancy of the UTAUT model.

Several limitations should be acknowledged when considering the results of our study. Our study sample was small. The pre-and post-learning evaluations only measured immediate, short-term effects. Repeat measures may be necessary to identify retention of learning and stabilization of critical thinking skills. The generalizability of our results is limited because most of the residents who participated were from the department of Anesthesiology. Due to the voluntary nature of residents' participation, selection bias cannot be excluded because more motivated residents were presumably more likely to enroll. Additionally, confounding factors might affect the internal validity of the results. For example, pain education before or during the intervention or enrollment of those with very little pain education could impact changes in scores. However, we excluded residents with formal pain education, and we limited the e-learning time to 7 days, which limited the available time for additional pain training. Finally, our study lacked a control group that underwent conventional didactic education on pain, and we cannot exclude an improvement in the pre-test assessment with repeated testing, particularly for the Critical Thinking test.

## Conclusions

The self-directed case-based e-learning platform in cancer pain management significantly improved knowledge and critical thinking in multidisciplinary residents. This e-learning platform might play an important role in pain education. Future studies should include larger numbers of participants from additional disciplines and compare the effectiveness of our platform to conventional didactics.

### Acknowledgements

The authors gratefully acknowledge the assistance of Ms. Wareeya Vongspanich for statistical analysis. Also, we thank the post-graduate medical department, including Mrs. Rujira Pethrak, for technical support. Lastly, we appreciated Prof. Christina Marra, Department of Neurology, University of Washington, USA, for English proofreading and generosity in manuscript editing.

### Conflict of Interest

The authors declare that they have no conflict of interest.
